# Antimicrobial Peptide Nanoassemblies: Design, Response Mechanisms, and Biomedical Applications

**DOI:** 10.3390/molecules31030518

**Published:** 2026-02-02

**Authors:** Tao Wang, Linbao Ji, Yucheng Zhang, Zhili Niu, Xiaoyi Jiang, Xingyao Wang, Qingtai Zhang, Yuting Zhang, Peng Tan, Yue Feng, Xi Ma, Zhihong Sun

**Affiliations:** 1 Research Center for Bio-Feed and Molecular Nutrition, College of Animal Science and Technology, Southwest University, Chongqing 400715, China; 2 State Key Laboratory of Animal Nutrition, College of Animal Science and Technology, China Agricultural University, Beijing 100193, China; 3 Key Laboratory of Chongqing Education Animal Nutrition and Bio-Feed of China, Chongqing 400715, China

**Keywords:** antimicrobial peptide nanoassemblies, self-assembly, intelligent response, molecular design, anti-infection

## Abstract

The overuse of antibiotics has accelerated the evolution and mutation of drug-resistant bacteria, creating an urgent need for novel antimicrobial drugs and feed additives. Antimicrobial peptides, with their unique membrane-disrupting mechanism that resists the development of resistance, hold promise as antibiotic alternatives. To overcome the limitations of natural antimicrobial peptides—such as poor stability, susceptibility to protease degradation, and short in vivo half-lives—self-assembling peptide technology has emerged. This approach employs non-covalent interactions to orderly assemble monomeric peptides into stable, structured nanomaterials like nanofibers, nanotubes, and hydrogels. This paper outlines the molecular design principles and smart response mechanisms of antimicrobial peptide nanoassemblies, elucidates their core advantages over monomeric peptides, summarizes their application scenarios in anti-infection fields, and discusses limitations and future directions across various domains. It provides insights for future antimicrobial peptide design.

## 1. Introduction

The excessive and unregulated use of antibiotics has disrupted the delicate balance between antimicrobial agents and bacterial resistance, accelerating the evolution and mutation of drug-resistant bacteria [1]. Mathematical models predict that over the next 25 years, more than 39 million people worldwide may die from infections caused by drug-resistant bacteria [2]. To address this global crisis, many countries and regions have implemented various policies to restrict the non-therapeutic use of antibiotics. For example, the Chinese government has issued a ban on the addition of antibiotics to animal feed [3]. Therefore, the search for safe, effective novel antimicrobial drugs and feed additives has become particularly crucial.

Antimicrobial peptides have attracted significant interest from researchers in biomedicine and animal husbandry due to their unique non-specific membrane penetration mechanism, which makes them resistant to the development of drug resistance [3,4,5,6,7,8,9,10,11,12]. Currently, dozens of peptide-based antimicrobial drugs have entered clinical trial phases, demonstrating promising application prospects [13]. However, it is important to note that monomeric antimicrobial peptides exhibit certain unavoidable limitations in practical applications. For instance, within biological systems, monomeric antimicrobial peptides are sensitive to environmental factors such as serum, salt ions [14], and proteases [15] (e.g., trypsin, pepsin, and chymotrypsin), leading to a significant reduction in their antimicrobial activity. Due to their small molecular weight, monomeric peptides are rapidly cleared by the kidneys, preventing the maintenance of effective therapeutic concentrations at the site of infection. Furthermore, treating localized infections with systemic or high-concentration antimicrobial peptides inevitably leads to off-target effects, causing unnecessary toxicity that compromises therapeutic efficacy [6].

**Figure 1:**
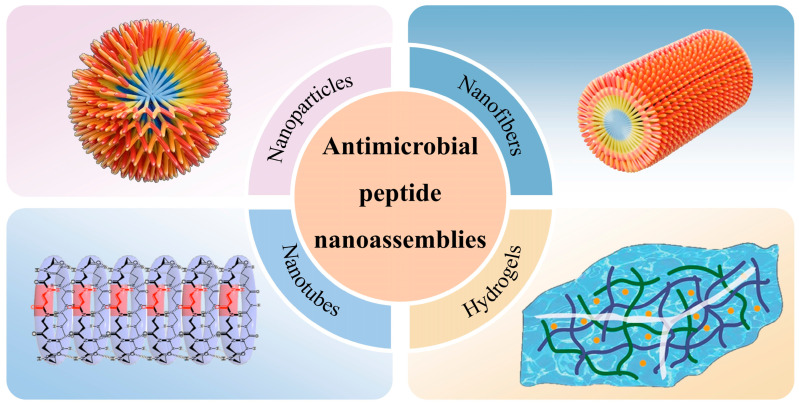
Schematic of the Four Morphologies of Self-Assembled Antimicrobial Peptide Nanostructures [3,19,20]. Copyright 2023, Wiley-VCH. Copyright 2023, Elsevier. Copyright 2025, Elsevier.

To address the limitations of natural antimicrobial peptides, the design of antimicrobial peptide nanoassemblies through self-assembly nanotechnology is considered a highly promising strategy (Figure 1). Compared to natural antimicrobial peptide molecules, antimicrobial peptide nanoassemblies can spontaneously organize into stable, ordered higher-order structures—such as nanofibers, nanotubes, and hydrogels—under the influence of non-covalent interactions (e.g., hydrogen bonding, hydrophobic interactions, and electrostatic forces) [16,17]. These higher-order structures enhance peptide stability by concealing functional regions of the antimicrobial peptides through physical barriers [18]. Self-assembly nanotechnology enables the design of peptides to aggregate and exert antibacterial activity specifically in the unique microenvironment of bacterial infections, thereby minimizing off-target damage to host cells [3]. In this review, we summarize the latest advances in the biomedical applications of self-assembled nanopeptides and discuss the technical challenges and future prospects of antimicrobial peptide nanotechnology.

## 2. Basic Characteristics and Design Strategies of Antimicrobial Peptide Nanoassemblies

Antimicrobial peptide nano self-assemblies refer to a class of functional molecular systems in which monomeric antimicrobial peptides, under specific environmental conditions, are driven synergistically by intermolecular non-covalent interactions—such as hydrogen bonding, π-π stacking, hydrophobic interactions, electrostatic forces, and van der Waals forces—to spontaneously form ordered nanostructures. Additionally, external system parameters such as pH, temperature, ionic strength, concentration, and solvent properties can also regulate these interactions [16]. When the system reaches dynamic equilibrium, the antimicrobial peptide molecules may become trapped in a metastable state and assemble, via a bottom–up approach, into various morphological nanostructures such as fibers, gels, nanospheres, and nanotubes [21]. Therefore, understanding these non-covalent interactions and their response mechanisms to external stimuli provides an important theoretical foundation for the rational design and preparation of antimicrobial peptide nano self-assemblies. This will be discussed in detail next.

### 2.1. Self-Assembly Mechanism

Hydrophobic interactions are one of the primary driving forces promoting the self-assembly of antimicrobial peptides. On the one hand, aliphatic amino acids such as leucine (L), isoleucine (I), and valine (V) provide non-directional hydrophobic forces through their nonpolar side chains. On the other hand, aromatic amino acids like phenylalanine (F), tryptophan (W), and tyrosine (Y) not only contribute hydrophobic effects but also offer directional π-π stacking interactions. When amphiphilic antimicrobial peptide molecules are in a polar environment (such as water), the hydrophobic regions tend to aggregate internally to form a hydrophobic core, minimizing the contact surface area between the molecules and the solvent, while the hydrophilic regions orient toward the polar environment. In addition to the driving force provided by conventional hydrophobic amino acids, alkyl chains of varying lengths (C4–C18), fatty acids such as palmitic acid (C16), and hydrophobic polymer segments (e.g., poly(lactic-co-glycolic acid) (PLGA)) are often introduced at the termini or side chains of antimicrobial peptides to enhance the self-assembly driving force [22,23,24,25,26,27].

Aromatic amino acids (phenylalanine F, tryptophan W, tyrosine Y) not only provide hydrophobic effects but also introduce directional π-π stacking interactions through their unique aromatic rings, thereby establishing a synergistic assembly mechanism [28]. The π-π stacking interaction is essentially an attractive force between the electron clouds of aromatic rings. Unlike the disordered structures formed by hydrophobic interactions, aromatic groups typically stack in specific spatial arrangements—either parallel or perpendicular—guiding antimicrobial peptide molecules into highly ordered alignments [29,30]. Moreover, π-π stacking often works in concert with other non-covalent forces such as hydrogen bonding and hydrophobic interactions, collectively enhancing the structural stability of self-assembling peptides. In addition to aromatic amino acids, many synthetic groups containing benzene rings can also provide π-π stacking. Examples include 9-fluorenylmethoxycarbonyl, 2-naphthylmethyl, naphthylalanine, pyrenylalanine, and anthrylalanine, among others [31,32,33,34,35,36].

Hydrogen bonding is also a crucial non-covalent force in driving self-assembly and protein folding [37]. The amide groups on the backbone and the hydroxyl, amino, and carboxyl groups on the side chains of antimicrobial peptides provide abundant hydrogen bond binding sites. In the *α*-helical conformation, hydrogen bonds can form between the carbonyl oxygen of an amide bond and the amide hydrogen of the fourth amino acid residue, running parallel to the direction of the peptide helix [38]. In the *β*-sheet conformation, adjacent peptide chains can form hydrogen bonds perpendicular to the peptide chain through interactions between amide groups [39]. Additionally, changes in external conditions such as pH and ionic strength can alter the protonation state of amino or carboxyl groups, thereby influencing hydrogen bond formation and breaking to achieve artificially controlled structural transitions in self-assembly [40,41]. Hydrogen bond-mediated secondary structures synergize with non-covalent interactions like hydrophobic forces and π-π stacking to guide peptide self-assembly into diverse ordered structures. Therefore, the precise design and regulation of hydrogen bonds constitute a crucial molecular foundation for constructing functionalized peptide-based self-assembled materials.

Hydrogen bonds frequently synergize with electrostatic interactions to drive self-assembly formation [42]. Electrostatic interactions fundamentally involve attractive or repulsive forces between charged amino acid residues. These primarily include positively charged residues like arginine, lysine, and histidine, and negatively charged residues such as aspartic acid and glutamic acid. On the one hand, electrostatic attraction between oppositely charged residues effectively drives peptide chains toward each other and promotes their ordered arrangement, forming specific oligomeric states or periodic structures. On the other hand, electrostatic repulsion between residues of the same charge effectively prevents excessive aggregation of peptide chains, maintaining the structure’s solubility and dynamic equilibrium. The combined action of these two electrostatic forces determines the size and morphology of self-assembled peptides [43]. Notably, changes in the external pH can alter the protonation state of amino acid residues, thereby reversing the net charge. Furthermore, altering ionic strength can influence peptide self-assembly by shielding electrostatic interactions [44,45,46,47]. Therefore, rational design of charged amino acid selection, quantity, and sequence arrangement during self-assembling peptide design enables the creation of dynamically functionalized antimicrobial peptide nanoassemblies.

### 2.2. Responsive Mechanisms

**Figure 2:**
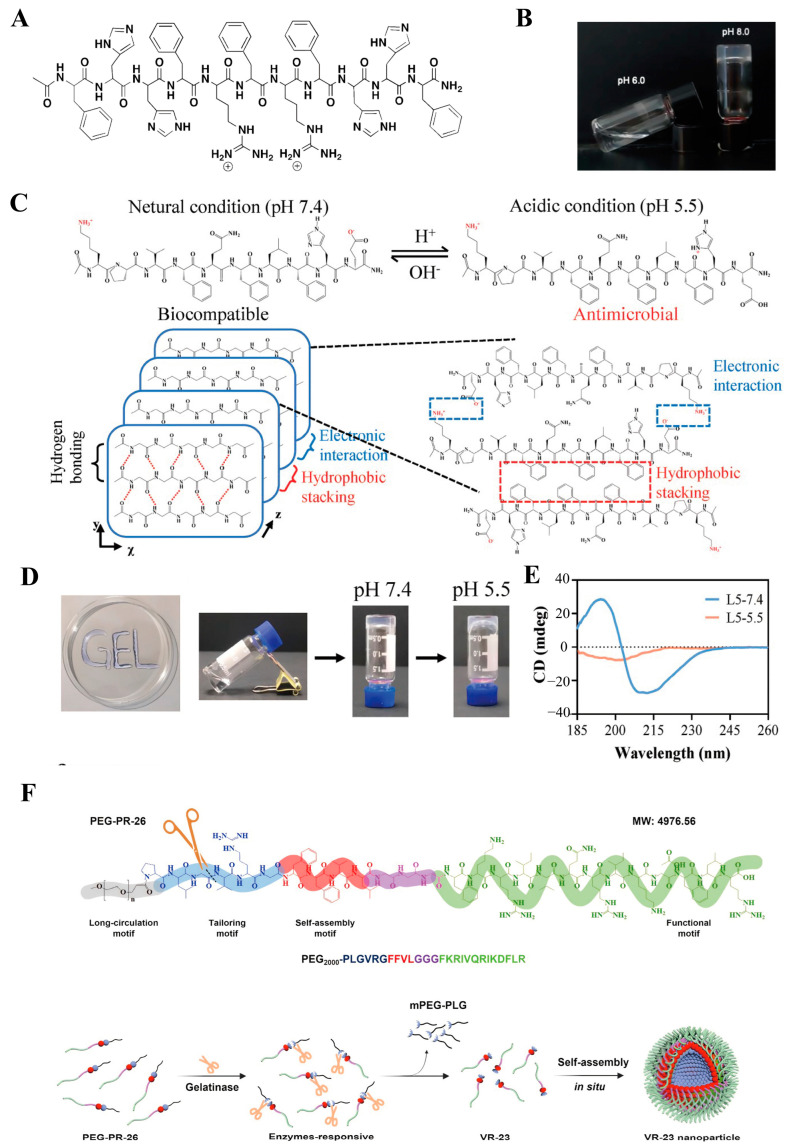
(**A**) Chemical structure of hydrogel FHHF-11. (**B**) At 37 °C, FHHF-11 remains liquid after incubation with a pH 6.0 buffer for 15 min, while it self-assembles into a hydrogel under the same conditions with a pH 8.0 buffer [50]. (**C**) At pH 7.4, L5 exhibits biocompatibility due to its electrically neutral state resulting from the combined effects of K and E residues. At pH 5.5, the protonation of the H residue side chain renders L5 positively charged, leading to the manifestation of antimicrobial activity. The intermolecular hydrogen bonding, electrostatic interactions, and hydrophobic packing of L5 at pH 7.4. (**D**) The “GEL” formed after injection of a 10 mg/mL L5 hydrogel using a needle with an inner diameter of 0.4 mm. The vial inversion method showed that after standing at room temperature for 0.5 h, L5 formed a stable solid gel at pH 7.4, but no solid gel formed at pH 5.5. (**E**) The CD spectra of L5 at different pH values [53]. Copyrights 2024, Wiley-VCH. (**F**) Schematic of the in situ self-assembled antimicrobial peptide PEG-PR-26 reacting to gelatinase. PEG-PR-26 releases the antimicrobial active molecule VR-23 under the action of gelatinase. VR-23 can rapidly undergo in situ self-assembly at the infection site in vivo and effectively kill pathogenic microorganisms. Reproduced with permission [56]. Copyrights 2025, American Chemical Society.

Compared to healthy tissues, the microenvironment of bacterial infections typically exhibits lower pH levels, elevated hydrogen peroxide concentrations, and excessive expression of enzymes and toxins [48]. These unique microenvironmental conditions can stimulate antimicrobial agents to achieve “on-demand” responsive release. Leveraging these properties, researchers frequently incorporate specific functional components to engineer antimicrobial peptide nanoassemblies that self-assemble into diverse structures upon stimulation, enabling tailored functions across varying physiological conditions [44]. During wound initiation, pH hovers around 5, gradually rising as healing progresses to reach approximately 8 upon complete resolution [49]. Leveraging this property, researchers designed the pH-responsive hydrogel FHHF-11 (Ac-FHHFRFRFHHF-CONH_2_) to exhibit varying stiffness across different pH levels, adapting to distinct wound healing stages [50]. Phenylalanine and histidine form the peptide core, with two additional arginine residues incorporated to enhance antimicrobial activity. At pH = 8, it forms a gel (G′ ≈ 50,000 Pa). Due to histidine’s pKa of 6, its imidazole side chain becomes protonated in acidic environments, forming a positively charged group that increases electrostatic repulsion and dissolves into a liquid state (G′ ≈ 0 Pa) (Figure 2A,B). Due to its excellent antibacterial activity and biocompatibility, it holds promise as a pH-responsive material with suitable properties for wound dressings.

Bacteria typically produce metabolites such as acetic acid, lactic acid, and malic acid at infection sites, creating an acidic environment with a pH ranging from 4.5 to 6.5 [51,52]. However, most antimicrobial peptide drugs exhibit reduced activity under such conditions. To address this, Liu et al. [53] designed a pH-responsive antimicrobial peptide nanoassembly L5 (Ac-KPVFQFLFHE-NH_2_) (Figure 2C) that functions specifically within the bacterial infection environment (pH 4.5–6.5). At pH = 7.4, the L5 peptide chain exhibits overall electroneutrality due to the combined action of positively charged lysine and negatively charged glutamic acid residues, forming a hydrogel under other intermolecular forces (Figure 2D). At acidic pH = 5.5, protonation of the histidine side chain causes dissolution of the self-assembled hydrogel and alteration of its secondary structure (Figure 2E), exhibiting excellent antibacterial activity against *Staphylococcus aureus* USA300. Endolysin LysSYL is a peptidoglycan hydrolase targeting staphylococci, and its enzymatic activity is easily compromised in acidic microenvironments. After encapsulation by L5, the resulting L5@LysSYL demonstrated significantly superior therapeutic effects compared to L5 alone at various treatment time points and exhibited sustained release properties.

In infection environments, bacteria often overexpress gelatinase, a type of matrix metalloproteinase that can rapidly degrade gelatin into peptides and amino acids. Leveraging this characteristic, many researchers have designed gelatinase-responsive materials capable of on-site triggered release, which serve as carrier materials for antibacterial drugs [54,55]. Based on the short peptide sequence PLGVRG that can be specifically cleaved by gelatinase, Yao et al. developed PEG-PR-26 (mPEG-PLGVRGFFVLGGGFKRIVQRIKDFLR) [56]. In *S. aureus*–infected areas, when exposed to gelatinase at the infection site, PEG–PR-26 is enzymatically cleaved to release the active fragment VR-23 (VRGFFVLGGGFKRIVQRIKDFLR), which contains the core antibacterial function of LL-37 (FKRIVQRIKDFLR) (Figure 2F). VR-23 rapidly self-assembles into nanoparticles (critical micelle concentration (CMC) = 2.31 μM) that disrupt bacterial membranes through localized depolarization and content leakage. Due to this transformation in activity and morphology triggered specifically by conditions in the infected area, not only is the antibacterial activity of LL-37 enhanced, but toxicity is also minimized. Even when a high dose (30 mg/kg) of PEG-PR-26 was injected into BALB/c mice, no adverse effects were observed.

**Figure 3:**
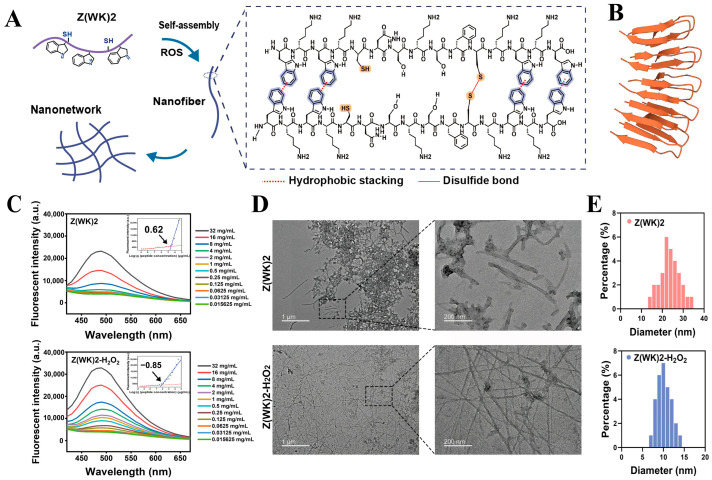
(**A**) Schematic diagram of Z(WK)_2_ self-assembly in ROS environment. (**B**) Predicted self-assembled structure of Z(WK)_2_. (**C**) 8-Anilino-1-naphthalenesulfonic acid (ANS) fluorescence intensity curves and Critical Aggregation Concentration (CAC) of Z(WK)_2_ with and without ROS. (**D**) TEM images of Z(WK)_2_ with and without ROS. (**E**) Diameter distribution of Z(WK)_2_ with and without ROS. Reproduced with permission [20]. Copyrights 2025, Elsevier.

Reactive oxygen species (ROS), a class of oxygen-derived chemical substances including the superoxide anion, hydroxyl radical, and hydrogen peroxide, play crucial roles in normal physiology and contributors to pathological processes. Common ROS primarily include superoxide anion, hydroxyl radical, and hydrogen peroxide. However, when ROS concentrations significantly increase within biological systems, they induce oxidative stress, damage cells, and trigger a series of pathological changes [57,58]. Capitalizing on the characteristic of significantly elevated ROS levels in pathological tissues, researchers have designed and synthesized various ROS-responsive antimicrobial peptide nanoassemblies. These structures not only enable in situ self-assembly for targeted action against pathogens but also alleviate oxidative stress by scavenging excess ROS within the body [59,60,61,62]. Meng et al. [20] designed an amphiphilic peptide Z(WK)_2_ (sequence: WKWKCNSKSFCKWKW) featuring alternating tryptophan and lysine residues at the C- and N-termini (Figure 3A,B). Within the high ROS microenvironment of Salmonella infection, amino acid residues in the peptide structure form intermolecular or intramolecular disulfide bonds to undergo in situ self-assembly (Figure 3C–E). This enhances both the ability to penetrate bacterial membranes and the targeted killing of pathogens in the lesion area.

In addition to the aforementioned environmental factors that can trigger peptide self-assembly responses, other conditions such as temperature, light stimulation, ionic reactions, and solvent interactions can also serve as triggers for antimicrobial peptide nanostructures to exert their antibacterial effects [44,45,63,64,65]. However, it is important to note that the infection microenvironment is often accompanied by complex physiological states. Relying solely on a single stimulus response may be insufficient to address the intricate conditions within a biological organism. Therefore, researchers have developed antimicrobial agents with dual-response or multi-response mechanisms to achieve more precise targeted bactericidal effects (Table 1).

During the laboratory design phase, integrating multiple responsive functional domains into antimicrobial peptide nanoassemblies to enable rapid and precise activation and drug release at infection sites remains a formidable challenge. Notably, most smart responsive antimicrobial peptide nanoassemblies remain confined to in vitro experiments. Ensuring their response accuracy and rapid drug release for effective biological treatment within the complex, dynamic in vivo environment—characterized by fluctuating enzymes, ion concentrations, and pH levels—will be a critical issue that future researchers cannot overlook.

### 2.3. Advantages of Antimicrobial Peptide-Based Nanoassemblies

**Figure 4:**
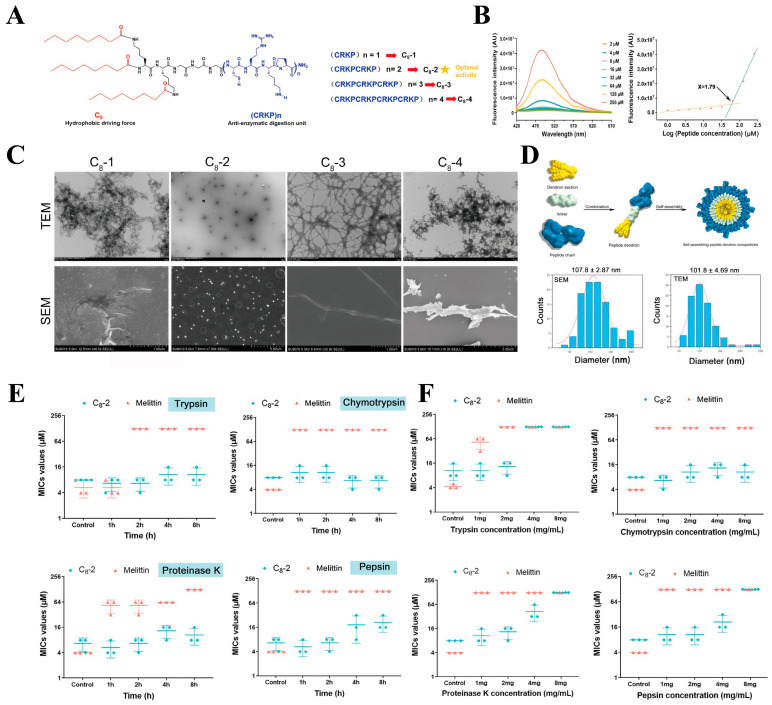
(**A**) Schematic diagram of C_8_-2 structure. (**B**) ANS fluorescence intensity curve of C8-2. (**C**) Transmission electron microscopy dark-stained and SEM images of the peptide dendrimer. (**D**) Schematic assembly diagram and diameter distribution of C_8_-2. (**E**) Effect of different incubation times with protease on the Minimum Inhibitory Concentration (MIC) of C_8_-2. (**F**) Effect of incubating C_8_-2 with protease at different concentrations for 8 h on its MIC. Reproduced with permission [82]. Copyrights 2025, Springer Nature.

**Figure 5:**
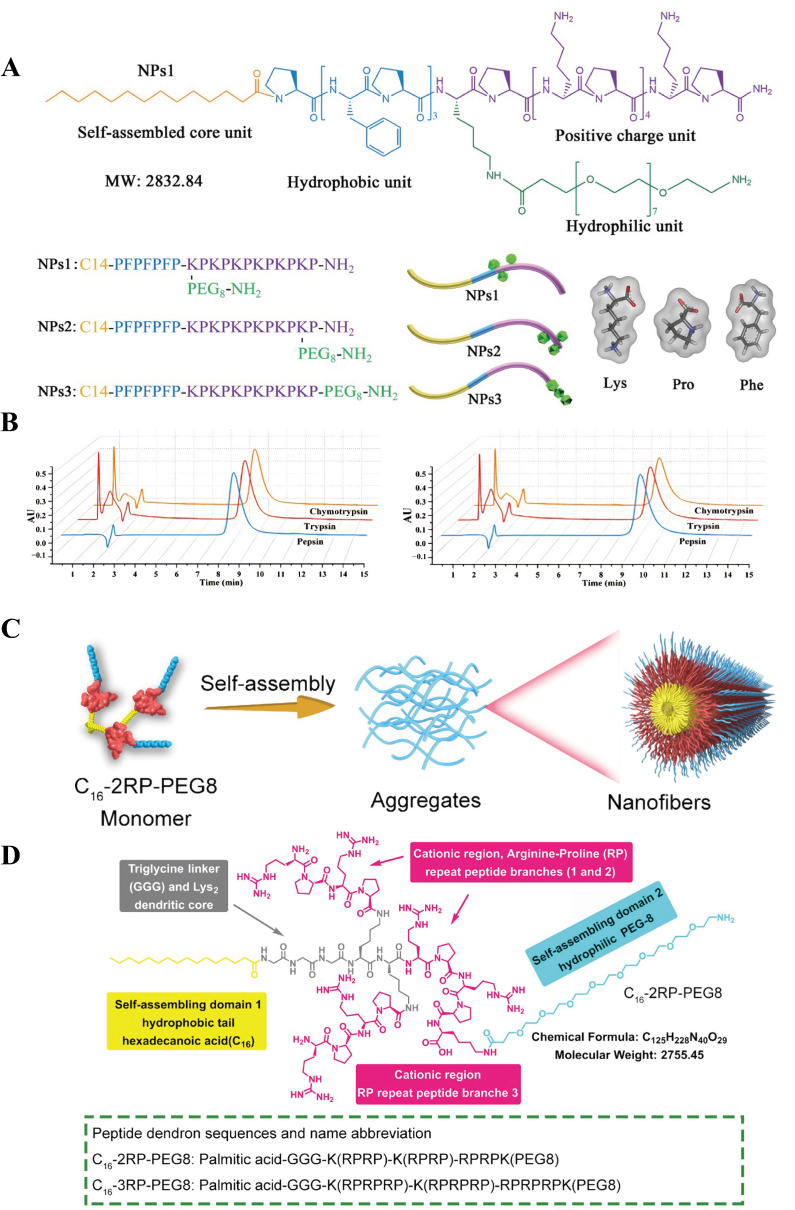
(**A**) Schematic diagram of the chemical structure of NPs. (**B**) RP-HPLC analysis of NPs1 and NPs2 after incubation with 8 mg/mL pepsin, trypsin, or chymotrypsin. Reproduced with permission [83]. Copyrights 2022, Wiley-VCH. (**C**) Schematic diagram of C_16_-2RP-PEG8 self-assembly. (**D**) Schematic diagram of the chemical structure of the peptide dendrimer. Reproduced with permission [84]. Copyrights 2024, Elsevier.

Antimicrobial peptides demonstrate broad application prospects in the biomedical field due to their high biological activity and excellent biocompatibility. However, within complex in vivo environments, peptide chains are highly susceptible to rapid degradation by various proteases, resulting in short biological half-lives and compromised therapeutic efficacy. To address this critical challenge, antimicrobial peptide nanoassemblies have emerged as a solution. When the concentration of antimicrobial peptides exceeds the CMC, they can form ordered higher-order structures through molecular self-assembly, thereby increasing the density of side chains and significantly shielding protease cleavage sites. This lays a solid foundation for advancing the practical application of antimicrobial peptides. Since chymotrypsin can specifically hydrolyze hydrophobic amino acids, Li et al. [82] selected fatty acids to provide hydrophobicity as the driving force for self-assembly and used the enzyme-resistant sequence CPKP to provide a positive charge. Subsequently, three glycine residues were employed as linkers to connect lysine with three fatty acids, resulting in self-assembling peptide dendrimer nanoparticles (Figure 4A–D). The peptide dendrimer C_8_-2 maintained excellent antifungal activity even after 8 h of enzymatic treatment (2 mg/mL trypsin, 8 mg/mL chymotrypsin, 2 mg/mL proteinase K, and 4 mg/mL pepsin) (Figure 4E). Furthermore, C_8_-2 retained effective antifungal activity even after 48 h of treatment with 100% human serum (Figure 4F). Our team has also contributed to the development of peptide-based self-assembling materials. Using C14 as both the driving force for self-assembly and a source of hydrophobicity, we placed proline residues on both sides of aromatic (phenylalanine) and cationic (lysine) amino acids to restrict cleavage by pepsin and trypsin. Finally, polyethylene glycol (PEG) was introduced at different positions along the peptide chain to further enhance the biocompatibility and enzyme resistance of the nanomaterials (Figure 5A) [83]. After co-incubation with proteases at a concentration of 8 mg/mL for 1 h, the peptide nanoparticles NPs1 and NPs2 maintained antimicrobial activity consistent with the control group. Remarkably, according to high-performance liquid chromatography (HPLC) analysis, even after extending the treatment time to 8 h, the peptide nanoparticles showed no significant degradation, demonstrating exceptional stability (Figure 5B).

A variety of strategies have been established to enhance the enzymatic stability of antimicrobial peptides, primarily including optimization of physicochemical properties, avoidance of enzymatic cleavage sites, chemical modifications to the peptide backbone, cyclization, incorporation of non-natural amino acids, and conjugation with protease inhibitors [15,85]. These approaches provide promising directions for the future clinical application of antimicrobial peptides. However, it is important to note that research on the actual efficacy of peptides within biological organisms, particularly in the gastrointestinal tract, remains limited. The vast majority of studies focus on stability testing under isolated in vitro conditions. The real in vivo environment often involves the combined effects of multiple factors, a scenario that cannot be overlooked. Furthermore, the increased costs associated with excessive chemical modifications of antimicrobial peptide nanostructures warrant careful consideration.

Compared to most synthetic polymers and inorganic materials, self-assembling peptides are predominantly composed of natural amino acids and fatty acids [86,87], inherently lacking the chemical toxicity and immunogenic risks associated with exogenous substances. Moreover, their primary metabolic byproducts in vivo are amino acids, which can be recycled by the body or participate in metabolic cycles. This prevents the accumulation of toxic substances and byproducts, reducing unnecessary inflammatory or foreign body reactions [88,89,90]. Under physiological conditions, self-assembling peptides spontaneously form higher-order structures. These structures closely mimic the biochemical properties of the natural extracellular matrix. As a result, they significantly mitigate cellular stress and reduce immune rejection responses [91]. The dendritic macromolecule C_16_-2RP-PEG8 (Figure 5C,D), designed via a branching synthesis approach with palmitic acid as the hydrophobic core and two repeating RP motifs for positive charge, exhibited no significant erythrocyte lysis even at 128 µM (32 times the MIC). In cytotoxicity assays, it maintained over 95% survival rate in Human Embryonic Kidney (HEK) 293T cells, demonstrating low cytotoxicity and excellent biocompatibility [84]. In contrast, the control bee venom peptide induced red blood cell rupture at very low concentrations. In in vivo biosafety evaluations, mice administered intraperitoneal injections of 10 and 20 mg/kg C_16_-2RP-PEG8 showed no significant differences from the saline control group across all parameters. Although reduced activity was observed in mice after 40 mg/kg injection, these effects resolved within 1 h. In contrast, the clinical drug polymyxin B resulted in 60% mortality within 2 h after intraperitoneal injection of 40 mg/kg in mice. Notably, the reduction in toxicity by self-assembled nanopeptides is not absolute. C_16_-3RP-PEG8, obtained by adding an additional RP repeat motif, exhibited marked cytotoxicity due to disruption of the optimal balance between functional domains. Collectively, these results demonstrate that rational design can achieve high safety in antibacterial peptide nanoassemblies, yet requires precise regulation of intramolecular interactions by researchers.

In addition to designing novel antimicrobial peptide nanoassemblies, modifying classic antimicrobial peptides into nanoformulations represents a crucial strategy for overcoming their inherent limitations and developing antimicrobial drugs. Gramicidin S (GS), the earliest discovered and most widely used cyclic antimicrobial peptide, exerts its bactericidal effect by targeting cell membranes. It has demonstrated potent antimicrobial activity during long-term clinical use and is less prone to inducing resistance. However, it is noteworthy that eukaryotic cell membranes are cholesterol-rich compared to bacterial membranes. When membrane cholesterol content reaches 30 mol%, GS exhibits its strongest binding affinity. Conversely, at 40 mol% cholesterol, its binding capacity diminishes. This leads to elevated toxicity toward certain eukaryotic cells, thereby limiting its systemic application [92]. Researchers modulated its membrane interactions by combining negatively charged star-shaped polyacrylamide copolymer (dextran-graft-polyacrylamide, D-g-PAA(PE)) with positively charged Bacillus thuringiensis toxin S. At concentrations of Gramicidin S below 8 mol%, D-g-PAA(PE) inhibits Gramicidin S binding to membranes. At 5 mol%, D-g-PAA(PE) effectively promotes Gramicidin S binding to the cell membrane in an oligomeric form, achieving precise regulation between antimicrobial activity and cytotoxicity [93].

## 3. Application of Antimicrobial Peptide Nanoassemblies in Anti-Infection

**Figure 6:**
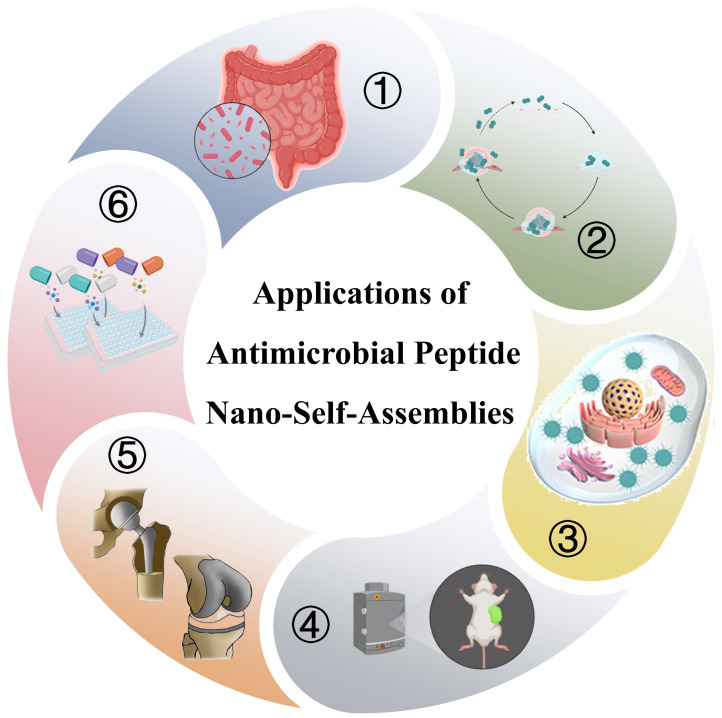
A schematic illustration of antimicrobial peptide nanoassemblies for combating bacterial infections. The graphic primarily includes their applications in ① the treatment of systemic bacterial infections, ② inhibition of biofilm formation and eradication of mature biofilms, ③ targeted clearance of intracellular infections, ④ construction of theranostic platforms, ⑤ anti-infective coatings for implantable devices, and ⑥ synergistic combination therapy with antibiotics [94,95]. Copyright 2025, Springer Nature. Copyright 2022, Elsevier.

Based on molecular design strategies and stimuli-responsive mechanisms, antimicrobial peptide nanoassemblies exhibit excellent biological activity. To translate these advantageous properties into clinically viable antimicrobial agents, researchers have engineered diverse functional nanoassemblies to combat microbial infections. As illustrated in Figure 6, their primary applications encompass several key areas: the treatment of systemic bacterial infections, inhibition of biofilm formation and eradication of mature biofilms, targeted clearance of intracellular infections, construction of theranostic platforms, development of anti-infective coatings for implantable devices, and synergistic combination therapy with antibiotics. This section will discuss the design principles, research progress, current limitations, and future directions within these domains.

### 3.1. Systemic Bacterial Infection Treatment

**Figure 7:**
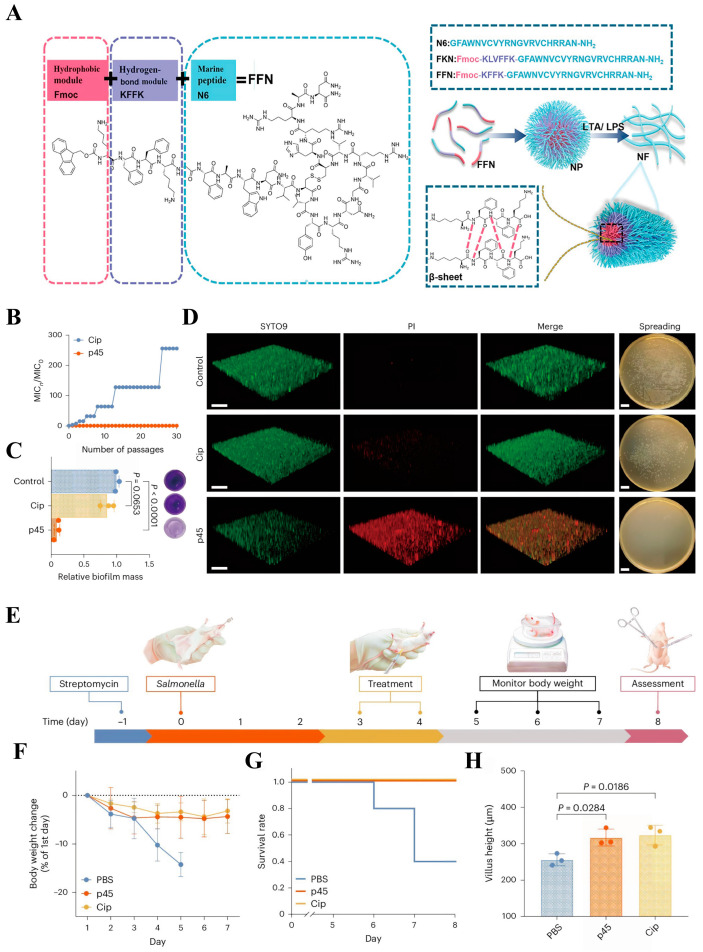
(**A**) Chemical structure of FKN and schematic diagram of self-assembly. Reproduced with permission [98]. Copyrights 2024, Springer Nature. (**B**) The fold change in the MIC of *Salmonella typhimurium* against ciprofloxacin and p45 was monitored over 30 serial passages. Here, MIC_0_ refers to the initial MIC value, while MIC_n_ denotes the value after *n* passages. (**C**) Relative mass and crystal violet staining images of *S. typhimurium* biofilms treated with PBS (control), Cip (50 × MIC), and p45 (50 × MIC). (**D**) Laser confocal microscopy images and brightfield micrographs of established *S*. *typhimurium* biofilms treated with Ciprofloxacin (Cip) and p45. (**E**) Experimental workflow diagram for mouse studies. Changes in body weight (**F**), survival rate (**G**), and mean villus length of the small intestine (**H**) in mice following treatment with PBS, p45, and ciprofloxacin (Cip). Reproduced with permission [103]. Copyrights 2025, Springer Nature.

The primary mechanism by which antimicrobial peptides exert their antibacterial effects involves electrostatic attraction between positively charged amino acid residues and negatively charged components on the pathogen membrane surface. This interaction induces the formation of amphiphilic structures that penetrate and permeabilize the bacterial phospholipid bilayer, leading to membrane depolarization and leakage of bacterial contents, ultimately resulting in bacterial death [96]. Additionally, antimicrobial peptides can self-assemble into various nanostructures through non-covalent interactions. This process further enhances the stability of antimicrobial peptide molecules, promotes membrane interactions, and modulates bacterial destruction mechanisms [97]. Lai et al. [84] constructed the peptide dendrimer C16-2RP-PEG8 by repeating the RP sequence twice and thrice. Following peritonitis–sepsis induction in mice via *Escherichia coli* HP73 infection, intraperitoneal injection of 5 mg/kg of the peptide dendrimer significantly reduced bacterial loads in the liver, kidneys, spleen, and lungs compared to controls. The natural marine peptide N6 (GFAWNVCVYRNGVRVCHRRAN-NH_2_) is a single peptide exhibiting high antimicrobial activity. Researchers selected Fluorenylmethyloxycarbonyl (Fmoc) as the hydrophobic module and designed KFFK based on Alzheimer’s disease amyloid protein Aβ40 (KLVFFAE) to provide hydrogen bonding and π-π stacking forces for the nanostructure, ultimately synthesizing FKN (Fmoc-KFFK-GFAWNVCVYRNGVRVCHRRAN-NH_2_) (Figure 7A). After 8 h of incubation in 25% serum, the MIC of template peptide N6 increased twofold, whereas the antibacterial activity of FKN remained unaffected under identical conditions. Furthermore, N6 was completely degraded after 4 h co-incubation with trypsin, whereas FKN retained 44.2% activity after 6 h co-incubation with trypsin and demonstrated significant therapeutic efficacy against *E. coli*/*S. aureus*-induced mouse mastitis [98].

However, most current research primarily focuses on in vitro cytotoxicity experiments, with in vivo studies predominantly using mice as experimental animal models. It remains unknown whether long-term use may lead to toxicity accumulation and associated pathological conditions. Additionally, after a series of chemical modifications, the degradation products and metabolic pathways of antimicrobial peptide molecules in vivo are still unclear. Future research should build upon this foundation to systematically evaluate the pharmacokinetics, tissue distribution, immune responses, and potential toxicity of antimicrobial peptide nanoassemblies in large animal models. Furthermore, integrating in vivo imaging and biosensing technologies would enable real-time monitoring of structural changes and therapeutic efficacy of these nanoassemblies within living animals.

### 3.2. Anti-Biofilm Strategies

Biofilms are structured microbial communities formed by microbial populations (including bacteria, fungi, algae, etc.) and their self-secreted adhesive substances (extracellular polymeric substances, EPSs), which attach to biological or non-biological surfaces [99]. Due to the presence of biofilms, various pathogenic microorganisms can survive even under harsh conditions, blocking the entry of most antimicrobial drugs to eliminate the pathogens inside or evading the host’s immune system [100,101]. Studies have shown that the presence of biofilms confers high levels of drug resistance to the bacteria within. Even when antibiotic concentrations are increased a thousandfold, conventional therapies often yield limited efficacy [102]. Antimicrobial peptide nanoassemblies possess strong penetration capabilities, ensuring the complete eradication of drug-resistant bacteria deep within biofilms. Based on the Database of Antimicrobial Activity and Structure of Peptides (DBAASP) and the deep learning (DL) model TransSAFP, Liu et al. [103] designed 140 self-assembling peptides, among which p45 exhibited the best antibacterial activity against multiple pathogens. p45 did not induce acquired resistance in continuous passage experiments with *S*. *typhimurium* (Figure 7B), and at a concentration of 50 × MIC, it could completely eradicate biofilms, outperforming ciprofloxacin in both aspects (Figure 7C,D). Moreover, in a mouse model of acute intestinal infection established by *S*. *typhimurium*, p45 demonstrated therapeutic efficacy comparable to that of ciprofloxacin (Figure 7E–H).

It is well known that biofilm formation primarily involves several stages: planktonic state, adhesion, colonization, maturation, and dispersal [104]. Therefore, effective intervention through anti-adhesion strategies at the initial stage of bacterial colonization to prevent biofilm formation is also a viable approach. Researchers have adopted an innovative “cocktail therapy”-inspired surface engineering design: by grafting nisin (which targets the cell wall precursor lipid II) onto antimicrobial peptide self-assembled monolayers, a dual-functionalized hybrid surface was constructed [105]. The results demonstrated that this hybrid surface not only retained the inherent contact-killing capability of Antimicrobial Peptide Self-Assembled Monolayers (AMP-SAMs) but also, due to the synergistic effect between nisin and AMPs (typically involving membrane lysis mechanisms), exhibited highly efficient and broad-spectrum antimicrobial activity far surpassing that of surfaces with single components. Particularly importantly, this “immediate and controllable” potent eradication effect on adhered bacteria significantly weakens the initial colonization capacity of bacteria at the root level, thereby offering a highly promising novel surface anti-adhesion strategy for effectively blocking biofilm formation from the initial stages.

**Figure 8:**
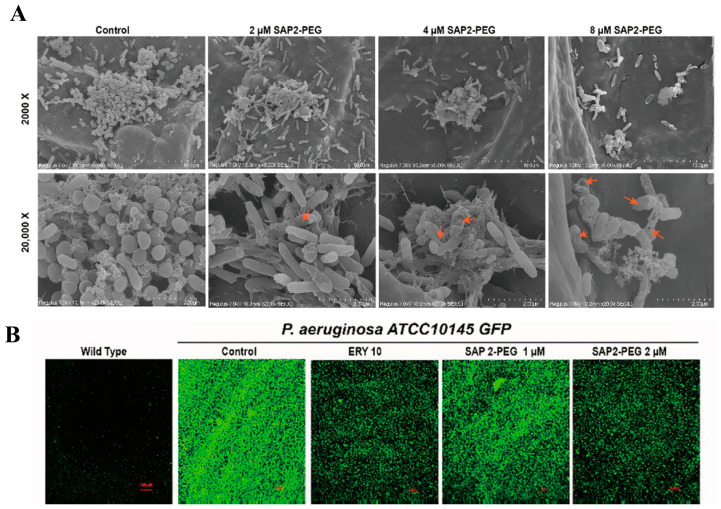
(**A**) SEM images of mature biofilms treated with different concentrations of SAP2-PEG. The arrows indicate that after treatment with 2, 4, and 8 μM SAP2-PEG, bacterial cell membrane damage and significant disruption of the biofilm structure are observed. Copyrights 2025, Wiley-VCH. (**B**) Confocal Laser Scanning Microscopy (CLSM) images of *P. aeruginosa* ATCC10145 GFP treated with erythromycin and SAP2-PEG (1 μM and 2 μM). Reproduced with permission [112]. Copyrights 2025, Wiley-VCH.

Quorum sensing is a communication system in bacteria, where microorganisms coordinate collective behavior by producing and detecting signaling molecules, playing a crucial role in biofilm formation and maintenance [106]. In Gram-positive and Gram-negative bacteria, biofilm formation is regulated by multiple interconnected circuits utilizing peptide-based quorum sensing systems and signals from N-acyl-homocysteine lactones and quinolones, respectively [107,108,109]. Consequently, targeting microbial quorum sensing systems presents an attractive therapeutic approach. Studies indicate that aromatic amino acids such as tryptophan and phenylalanine can disrupt microbial quorum sensing systems [110,111]. Strategically incorporating these amino acids during the design of self-assembling peptides can effectively disrupt biofilm formation. Based on this, researchers designed SAP2-PEG, a self-assembling peptide comprising three segments: aromatic amino acids tryptophan and phenylalanine to provide hydrophobic interactions; lysine and arginine to enhance binding to negatively charged microbial components; and PEG modification at the C-terminus to improve enzymatic stability and balance amphiphilic properties [112]. Compared to the control group, SAP2-PEG effectively penetrated and disrupted the extracellular polymers of biofilms (Figure 8A), demonstrating superior efficacy to gentamicin in inhibiting biofilm formation and clearing mature biofilms. Treatment at subinhibitory concentrations not only effectively suppressed the green fluorescent signal of *Pseudomonas aeruginosa* ATCC 10145 (QS report strain) to a degree comparable to 10 μM erythromycin (Figure 8B), but also significantly reduced the production of associated virulence factors. Following treatment with SAP2-PEG at 0.65 times the MIC, *P. aeruginosa* growth capacity remained unaffected, while both motility and swarming movements were severely inhibited.

Within mature biofilms, parameters such as pH, oxygen levels, bacterial activity, and the composition and density of extracellular polymeric substances exhibit heterogeneous distribution. Current research on the responsive mechanisms of antimicrobial peptide nanoassemblies is mostly conducted under idealized experimental conditions. In complex and dynamically changing biofilm environments, whether these nanoassemblies can be precisely activated in targeted regions and release drugs accurately remains to be investigated. Moreover, most existing studies still rely on 96-well plate models to evaluate the inhibitory and eradication effects of antimicrobial peptide nanoassemblies on biofilms. Such experimental results often cannot serve as reliable preclinical evidence. Furthermore, most experimental results indicate that antimicrobial peptide nanoassemblies cannot completely eradicate biofilms. Therefore, future research should focus on integrating multiple mechanisms to synergistically disrupt biofilm structures. Beyond eliminating established biofilms, developing anti-fouling coatings based on antimicrobial peptide nanoassemblies to prevent biofilm colonization during its initial stages represents a highly promising strategy.

### 3.3. Treatment of Intracellular Bacterial Infections

Intracellular bacterial infections, due to the host cell’s unique pH, redox potential, and enzymatic activity, result in poor membrane permeability and low intracellular stability of conventional antibacterial drugs, leading to intracellular drug concentrations insufficient to achieve bactericidal effects [113]. For example, β-lactam and aminoglycoside antibiotics exhibit limited cellular penetration and cytoplasmic accumulation within host cells due to their hydrophilic nature. In contrast, fluoroquinolone and macrolide antibiotics demonstrate poor intracellular retention after entering host cells [114]. While increasing the dosage and extending the treatment duration may improve this situation, it may be accompanied by adverse effects such as the disruption of intestinal microbial homeostasis, accelerated drug resistance, and unnecessary toxicity and allergic reactions [115,116,117,118].

**Figure 9:**
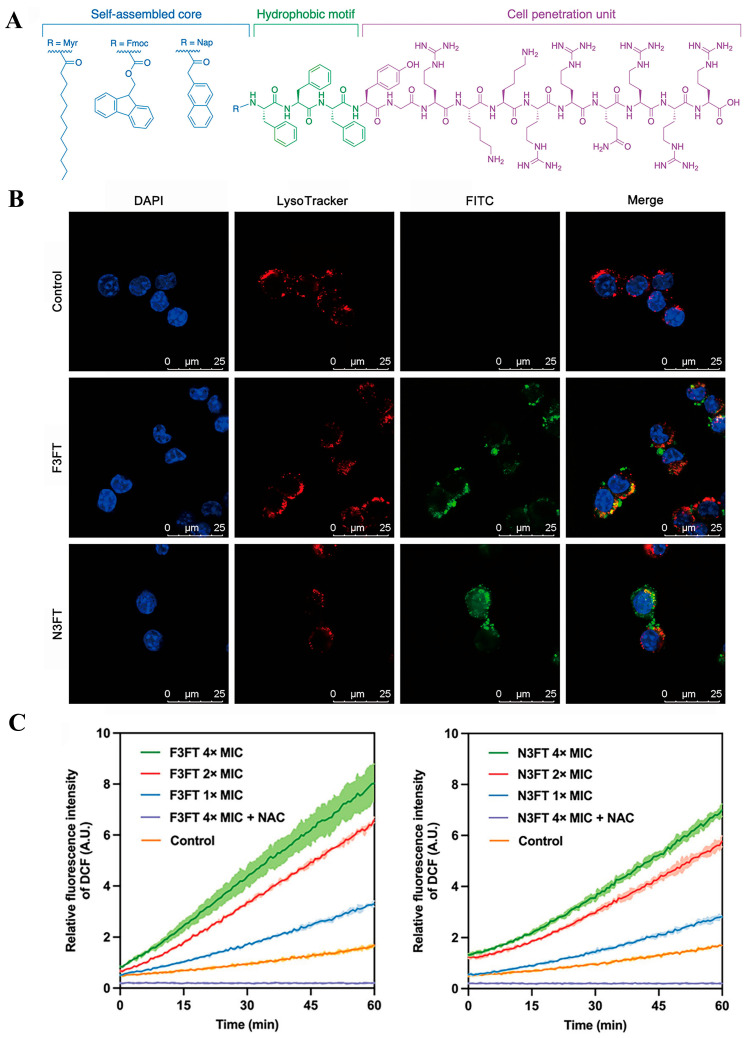
(**A**) Chemical structures of M3FT, F3FT, and N3FT. (**B**) Using the fluorescent probes 4′,6-diamidino-2-phenylindole (DAPI) and LysoTracker as indicators, the intracellular localization of Fluorescein Isothiocyanate (FITC)-labeled F3FT and N3FT was observed by confocal laser scanning microscopy. (**C**) The relative fluorescence signal intensity accumulated over time by *Staphylococcus aureus* 25923 after treatment with different concentrations of F3FT and N3FT. DCF, 2′,7′-Dichlorodihydrofluorescein diacetate. NAC, N-Acetyl-Cysteine. Reproduced with permission [94]. Copyrights 2025, The American Association for the Advancement of Science.

Cell-penetrating peptides (CPPs) are short peptides consisting of 5–30 amino acids with high cell-penetrating efficiency. They can transport many biomacromolecules (such as proteins, nucleic acids [119], and even nanomaterials) across the plasma membrane into various cells and tissues, offering significant potential for drug delivery and gene therapy. Currently, many studies have used CPPs to deliver antibiotics against intracellular bacterial infections [120,121], but the bacterial resistance induced by this strategy could become a more troublesome issue. Coupling CPPs with antimicrobial peptide nanoassemblies is a promising idea. Zhu et al. [94] selected Fmoc and naphthalene (Nap) at the N-terminus to drive self-assembly, placed the highly positively charged CPP transcription trans-activator (Tat, YGRKKRRQRRR) at the C-terminus to attract negatively charged bacterial membranes and facilitate cell penetration, and finally added three phenylalanines as a hydrophobic motif between them to further enhance the affinity of the antimicrobial peptide nanoassemblies for bacteria (Figure 9A). The designed F3FT (Fmoc-FFFYGRKKRRQRRR) and N3FT (Nap-FFFYGRKKRRQRRR) could self-assemble into nanoparticles and nanofibers at concentrations of 21.35 and 34.91 μM, respectively. Research results showed that F3FT and N3FT could not only penetrate bacterial membranes via macropinocytosis but also induce excessive accumulation of reactive oxygen species (Figure 9B,C). Notably, in a mouse peritonitis sepsis model infected with *S. aureus*, F3FT and N3FT significantly reduced the bacterial load in various organs and peritoneal macrophages, with effects far superior to vancomycin.

Although antimicrobial peptide nanoassemblies can combat intracellular infections by coupling with cell-penetrating peptides, excessively enhanced membrane penetration or destructive mechanisms may damage host cells, raising safety concerns. How to ensure antimicrobial efficacy while minimizing toxicity to host cells warrants consideration. Furthermore, relying solely on a single membrane lysis mechanism may allow bacteria to develop tolerance by adjusting membrane composition or activating stress responses. Future approaches could employ modular design, utilizing antimicrobial peptide nanoassemblies as carriers to deliver other drugs into infected cells for synergistic treatment of intracellular bacterial infections.

### 3.4. Implant Coating

In modern medical practice, with the widespread use of implants, implant-associated infections have become a significant challenge in clinical treatment [122,123]. Statistics indicate that over 14 million hospital-acquired infections annually are caused by implant-related infections. Since implant surfaces are susceptible to bacterial adhesion and colonization during the first four weeks post-implantation, early prevention is particularly crucial [124]. Given the limited efficacy of traditional oral and intravenous administration methods for treating implant infections, coupled with their potential to impose significant burdens on the biological organism [125], researchers are dedicated to developing implant devices with inherent antimicrobial capabilities by covalently fixing antimicrobial agents to their surfaces.

Yao et al. [126] developed an antibacterial hydrogel coating based on the Schiff base reaction, utilizing FK-13 (sequence: FKRIVQRIKDFLR)—the core region responsible for LL-37’s antimicrobial activity—as the active agent. This coating was designed to prevent infections associated with cardiovascular implantable electronic devices. This system leverages the Schiff base bond formed between hyaluronic acid-nitrobenzene (HN) and FK-13 to maintain the peptide’s biological activity. Consequently, it ensures sustained release of the therapeutic peptide FK-13 without requiring any external stimuli. In an in vivo subcutaneous implantation model, the PN-FK hydrogel demonstrated robust anti-infection and tissue regeneration capabilities. Compared to bare titanium implants, this system significantly reduced inflammatory infiltration induced by metal sheet implantation and promoted the generation of collagen and blood vessels, showing great potential in preventing cardiovascular implantable electronic device-related infections. Liu et al. [127] constructed a composite coating composed of a fusion peptide integrating an antimicrobial peptide (HHC36) and an angiogenic peptide on the surface of 3D-printed porous tantalum (Ta-CCS@FP) through alkali treatment, electrostatic adsorption, and EDC/NHS reactions. Compared to conventional peptide mixtures, the fusion peptide approach, which combines peptides of different sequences into a single polypeptide chain, effectively addresses the limitations of grafting site availability and spatial/directional control during the grafting process of peptide mixtures while preserving their respective bioactivities [128]. In both in vitro and cellular experiments, this functionalized implant demonstrated potent antibacterial and anti-biofilm properties, as well as osteogenic and angiogenic capabilities, due to the synergistic effect between the HHC36 peptide and the carboxymethyl chitosan (CCS) coating. The in vivo infectious bone defect model further confirmed that Ta-CCS@FP could effectively eliminate pathogenic bacteria within two weeks and promote the regeneration of blood vessels and bone tissue within six weeks. This study provides a comprehensive treatment strategy for porous tantalum implants that integrates anti-infective properties with enhanced osseointegration capabilities.

Under the long-term and complex physiological conditions within the body, implant coatings may gradually lose efficacy due to wear, enzymatic degradation, or protein adsorption. Additionally, balancing the dual functions of antimicrobial activity and promoting host cell proliferation in implant coatings presents a significant challenge. Furthermore, most implant coatings currently remain at the laboratory stage, making the establishment of standardized, scalable processes a critical consideration for the future.

### 3.5. Diagnosis and Treatment Integration Platform

Traditional anti-infection strategies primarily involve post-diagnosis treatment. Due to their shortcomings in timeliness and precision, there is an urgent need to optimize therapeutic approaches. Against this backdrop, integrated diagnosis-and-treatment strategies have emerged. Such strategies enable real-time, dynamic monitoring of pathogenic microorganisms during the therapeutic process, making anti-infection technologies more precise, dynamic, and efficient. At the design stage, signal-reporting functional modules (such as fluorescence, magnetic resonance, and acoustic response modules) can be integrated into antimicrobial peptide nanoassemblies, thereby creating an integrated platform for diagnosis and treatment [129,130,131].

**Figure 10:**
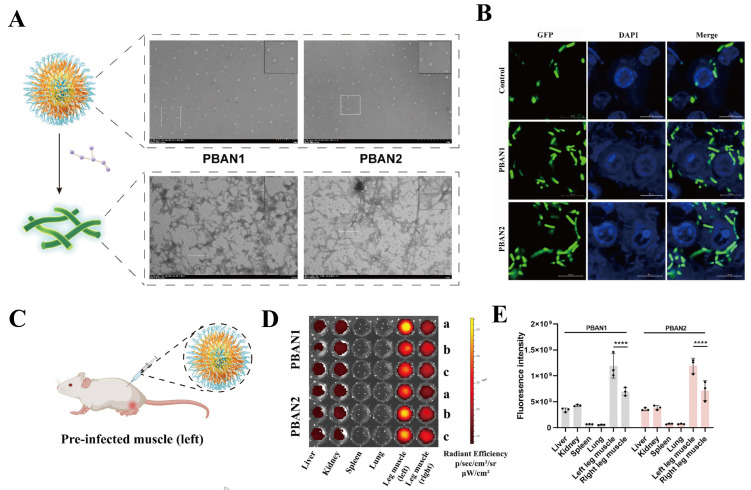
(**A**) In the absence of LPS, Transmission Electron Microscopy (TEM) imaging revealed that PBANs self-assembled into nanoparticles. Conversely, upon LPS treatment, TEM showed that PBANs self-assembled into nanofibers. The insets represent enlarged views of the corresponding regions. (**B**) Confocal laser scanning microscopy was used to assess the phagocytosis of Green Fluorescent Protein (GFP)-labeled *E. coli* by RAW 264.7 cells. GFP, GFP-labeled *E. coli*; Diamidino-2-phenylindole (DAPI), DAPI-stained RAW 264.7 cells. (**C**) Schematic diagram of mouse thigh infection model. (**D**) Fluorescence images of the liver, kidneys, spleen, lungs, left hind leg, and right hind leg in mice after injection of PBANs. a, b, and c represent three repetitions. (**E**) Quantitative fluorescence signal intensity from the liver, kidneys, spleen, lungs, left hind leg, and right hind leg in mice after injection of PBANs. (**** *p* < 0.0001). Reproduced with permission [132]. Copyrights 2025, Wiley-VCH.

Our team incorporated a tetraphenylethylene (TPE) functional module into the antimicrobial peptide [132]. On the one hand, this provides a driving force for peptide self-assembly; on the other hand, due to its aggregation-induced emission (AIE) property, it can be used for fluorescent signal reporting. Subsequently, the peptide was connected via three phenylalanines to HVF18 (HVFRLKKWIQKVIDQFGE), which has a strong affinity for the bacterial cell wall component Lipopolysaccharide (LPS). Finally, cationic amino acid arginine was introduced at the C-terminus to further enhance electrostatic interactions with bacterial membranes, leading to the design and development of the aggregation-induced emission self-assembling peptides PBAN1 and PBAN2. Under physiological conditions, PBAN1 and PBAN2 self-assemble into nanoparticles. However, upon interacting with LPS on the bacterial surface, the peptides are induced to transform into nanofibers that surround and entangle the bacteria, restricting their mobility and preventing their invasion of the host system (Figure 10A). Furthermore, PBAN1 and PBAN2 can recruit macrophages to the infection site to phagocytose the trapped bacteria, thereby synergistically reducing infection efficiency (Figure 10B). Most notably, in both mouse and piglet systemic infection models, PBANs effectively labeled bacteria and suppressed bacterial infection in *E. coli* induced sepsis infection models. Influenced by different environmental factors, PBANs undergo a transformation in nanostructure, which further induces variations in the intensity of the fluorescent signal, thus providing a reference direction for integrated anti-infection diagnosis and treatment (Figure 10C–E).

However, after covalent conjugation of antimicrobial peptide nanoassemblies with imaging modules, whether the peptide’s conformation, self-assembly behavior, and bactericidal efficacy will be affected warrants attention. Additionally, in animal models, spontaneous fluorescence from tissues and fur, along with some nonspecific adsorption, may severely interfere with the signal-to-noise ratio of imaging. Moreover, the current lack of standardized models and methods makes it difficult to compare performance across different studies, hindering the identification of optimal design strategies and clinical advancement. In the future, constructing multimodal synergistic therapeutic platforms could enable comprehensive detection of infection sites.

### 3.6. Combination Antibiotic Therapy

Combination therapy has now emerged as a promising antimicrobial strategy. Due to their unique membrane-permeating bactericidal mechanism, antimicrobial peptide nanoassemblies represent a candidate for combination therapy with traditional antibiotics [133]. Antimicrobial peptide molecules can disrupt the integrity of bacterial membranes, enabling antibiotics to reach their target sites more efficiently [134]. Furthermore, antimicrobial peptide molecules can inhibit or eliminate microbial biofilms, exposing bacteria that would otherwise be protected by biofilms and thereby enhancing antibiotic killing of pathogens [135]. Additionally, antimicrobial peptides can directly enhance the efficacy of traditional antibiotics by altering bacterial metabolic processes or suppressing antibiotic resistance mechanisms, thereby increasing antibiotic susceptibility [136,137]. Therefore, the combination of antimicrobial peptides and antibiotics can significantly reduce drug dosage while improving therapeutic outcomes, making it a common strategy for treating multidrug-resistant bacterial infections. YS12 is an antimicrobial peptide isolated from *Bacillus cereus* CBSYS12 that exhibits antibacterial activity against multiple drug-resistant bacteria [138]. YS12 demonstrates a significant synergistic effect with ciprofloxacin against *E. coli* KCTC 1923 (Fractional Inhibitory Concentration Index (FICI) = 0.157). Moreover, the combination of YS12 with either ciprofloxacin or erythromycin also shows synergistic effects against *E. coli* KCTC 1923 and *S. aureus* KCTC 1928 (FICI < 0.5). However, YS12 exhibits an additive effect with ciprofloxacin against *P. aeruginosa* KCTC 1637 (FICI = 0.749) and even an antagonistic effect when combined with erythromycin against *P. aeruginosa* KCTC 1637 (FICI = 1) [139]. Furthermore, due to differences in the pharmacokinetics of different drugs in vivo, two antimicrobial agents that show synergy in vitro may perform poorly in vivo. To address this issue, researchers often chemically conjugate the two drugs to overcome this challenge [140].

MSI-78 is an antimicrobial peptide isolated from the skin secretions of the African clawed frog, which exerts its antibacterial effect by disrupting membranes [141]. Through chemical modification, a sulfur-containing amino acid cysteine was linked to the N-terminus of MSI-78, followed by conjugation with vancomycin via the linker sulfo-SMCC to obtain Vm-MSI [142]. Compared with vancomycin, the antibacterial activity of Vm-MSI was enhanced 20.18-fold. In addition to killing VRSA at extremely low concentrations, Vm-MSI also exhibited excellent broad-spectrum efficacy against many clinically drug-resistant Gram-negative bacteria. Moreover, Vm-MSI could effectively inhibit biofilm formation by VRSA, *E. coli*, and MRSA, and clear mature biofilms. In a VRSA-constructed skin infection model, Vm-MSI effectively reduced the bacterial load at the wound site, suppressed the expression of TNF-α and IL-6, and enabled complete skin wound healing 12 days after infection, with results comparable to those of the ciprofloxacin treatment group. Vancomycin, by contrast, showed unsatisfactory therapeutic effects. Furthermore, Vm-MSI also demonstrated outstanding therapeutic efficacy in a lung infection model constructed with multidrug-resistant *Acinetobacter baumannii*.

Due to the vast number of possible combinations between antimicrobial peptides and antibiotics, most of which are ineffective, traditional screening methods are time-consuming and have an excessively low success rate, greatly limiting the development speed of combination therapies. In the future, researchers could utilize artificial intelligence to conduct in-depth analysis of existing data on the combined application of antimicrobial peptides and antibiotics and predict self-assembling peptide sequences and antibiotic combinations with developmental potential, thereby improving research and development efficiency.

## 4. Conclusions

Antimicrobial peptide nanoassemblies hold promise as potential antimicrobial agents against bacterial drug-resistant infections due to their excellent biocompatibility and functional diversity. Through rational molecular design, researchers have constructed smart antimicrobial molecules capable of responding to multiple stimuli such as pH, enzymes, and ROS. These assemblies play a significant role in achieving targeted delivery of antimicrobial drugs, inhibiting and eliminating biofilms, and enabling real-time imaging at infection sites. Furthermore, synergistic applications across other disciplines have broadened the scope of antimicrobial peptide nanoassemblies. It is important to note that most current research on these assemblies remains confined to the laboratory stage, with limited progress into clinical trials. Future research should prioritize evaluating the actual efficacy of antimicrobial peptide molecules in complex in vivo environments, conducting long-term and systematic biosafety assessments, and exploring methods to reduce production costs for scalable manufacturing. Through multidisciplinary integration and technological innovation, antimicrobial peptide nanoassemblies hold promise as a new generation of smart antimicrobial molecules.

## Figures and Tables

**Table 1:** Representative Responsive antimicrobial Peptide Nanoassemblies.

Type	Peptide	Response	Application	References
Single Response	h-(P)-(M)-DMMA	pH	Antibacterial hydrogel vascular graft	[66]
Single Response	GE33	pH	Treatment of *Helicobacter pylori* infection	[67]
Single Response	Ti-NTs-P-A	pH	Treatment of *S. aureus*-induced osteomyelitis	[68]
Single Response	VCM-Na-Alg/AMP	pH	Targeted delivery of vancomycin and AMPs to combat MRSA	[69]
Single Response	AMP/VPM/PEG	Collagenase	Prevention of MRSA-induced osteomyelitis	[70]
Single Response	MCIP/(PEG-PCL)/PLL NPs	Lipase	Treatment of chronic pulmonary infection caused by multidrug-resistant *Pseudomonas aeruginosa*	[71]
Single Response	amino-SBA-15-SOAP@BSA	Trypsin	Treatment of local bacterial infections	[72]
Single Response	SFMA/rColMA/LNP@AMP@PUE	ROS	Antibacterial, anti-inflammatory, and pro-angiogenic effects to accelerate diabetic wound healing	[73]
Single Response	SS-hPG	GSH or DTT	Improve wound healing outcomes	[74]
Single Response	ACT	Light (660 nm laser, 0.8 W/cm^2^)	Targeted killing of *S. aureus* by combining bacterial capture with photodynamic therapy	[75]
Single Response	W379, IR780 iodide and PVP	NIR light (0.4 W/cm^2^)	Drug release to combat biofilms and promote wound healing	[76]
Single Response	TPI-CysHHC10	White light (60 mW/cm^2^)	Treatment of MRSA-induced wound infection	[77]
Single Response	CMC-DES5	Temperature	Intelligent real-time monitoring and treatment of traumatic infections, and diabetic wound healing	[78]
Dual Response	VCM-AMP-TF-NLC	Lipase and Matrix Metalloproteinase	Bacterial infection	[79]
Dual Response	SA/FK13-a1@GelMA MNs	pH and Matrix Metalloproteinase	Treatment of tinea pedis or other fungal skin diseases	[80]
Dual Response	MN/DPP@AC	pH and light	Elimination of biofilms and treatment of stalled healing in chronic wounds	[81]

## Data Availability

No new data were created or analyzed in this study. Data sharing is not applicable to this article.
